# Alpha-Glucosidase Inhibitory Effect of *Psychotria malayana* Jack Leaf: A Rapid Analysis Using Infrared Fingerprinting

**DOI:** 10.3390/molecules25184161

**Published:** 2020-09-11

**Authors:** Tanzina Sharmin Nipun, Alfi Khatib, Qamar Uddin Ahmed, Irna Elina Redzwan, Zalikha Ibrahim, Al’aina Yuhainis Firus Khan, Riesta Primaharinastiti, Shaden A. M. Khalifa, Hesham R. El-Seedi

**Affiliations:** 1 Pharmacognosy Research Group, Department of Pharmaceutical Chemistry, Kulliyyah of Pharmacy, International Islamic University Malaysia, Kuantan 25200, Pahang Darul Makmur, Malaysia; tsn.np99@gmail.com (T.S.N.); quahmed@iium.edu.my (Q.U.A.); elina@iium.edu.my (I.E.R.); zalikha@iium.edu.my (Z.I.); 2 Department of Pharmacy, Faculty of Biological Sciences, University of Chittagong, Chittagong 4331, Bangladesh; 3 Faculty of Pharmacy, Airlangga University, Surabaya 60155, Indonesia; 4 Department of Biomedical Sciences, Kulliyyah of Allied Health Sciences, International Islamic University Malaysia, Kuantan 25200, Pahang Darul Makmur, Malaysia; alainayuhainis@gmail.com; 5 Department of Molecular Biosciences, The Wenner-Gren Institute, Stockholm University, SE-106 91 Stockholm, Sweden; hesham.elseedi@su.se; 6 Department of Chemistry, Faculty of Science, Menoufia University, Shebin El-Kom 32512, Egypt; 7 International Research Center for Food Nutrition and Safety, Jiangsu University, Zhenjiang 212013, China

**Keywords:** *Psychotria malayana*, α-glucosidase inhibition, orthogonal partial least square, fingerprint, infrared spectroscopy analysis

## Abstract

The plant *Psychotria malayana* Jack belongs to the Rubiaceae family and is known in Malaysia as “meroyan sakat/salung”. A rapid analytical technique to facilitate the evaluation of the *P. malayana* leaves’ quality has not been well-established yet. This work aimed therefore to develop a validated analytical technique in order to predict the alpha-glucosidase inhibitory action (AGI) of *P. malayana* leaves, applying a Fourier Transform Infrared Spectroscopy (FTIR) fingerprint and utilizing an orthogonal partial least square (OPLS). The dried leaf extracts were prepared by sonication of different ratios of methanol-water solvent (0, 25, 50, 75, and 100% *v*/*v*) prior to the assessment of alpha-glucosidase inhibition (AGI) and the following infrared spectroscopy. The correlation between the biological activity and the spectral data was evaluated using multivariate data analysis (MVDA). The 100% methanol extract possessed the highest inhibitory activity against the alpha-glucosidase (IC_50_ 2.83 ± 0.32 μg/mL). Different bioactive functional groups, including hydroxyl (O-H), alkenyl (C=C), methylene (C-H), carbonyl (C=O), and secondary amine (N-H) groups, were detected by the multivariate analysis. These functional groups actively induced the alpha-glucosidase inhibition effect. This finding demonstrated the spectrum profile of the FTIR for the natural herb *P. malayana* Jack, further confirming its medicinal value. The developed validated model can be used to predict the AGI of *P. malayana*, which will be useful as a tool in the plant’s quality control.

## 1. Introduction

The long-term metabolic disease produced by hyperglycemia is known as diabetes mellitus (DM), referring to a condition where a consistently high blood sugar level leads to an imbalance of tissue homeostasis. Various complications may develop if diabetes is not well managed; for instance, it can cause cardiovascular diseases and stroke [1]. Myopia, glaucoma, and retinal detachment may be caused by diabetic retinopathy [1]. The World Health Organization (WHO) has categorized three main variants of diabetes mellitus and identified it as a heterogeneous metabolic disease with high blood sugar [2]: type-1, insulin-dependent diabetes (T1DM); type-2, non-insulin-dependent diabetes (T2DM); and a third, gestational diabetes mellitus (GDM).

T2DM predominantly affects obese adolescent children and has been observed more in older individuals [2]. T2DM represents most of the cases worldwide [1]. The WHO had identified several T2DM risk factors, including genetics, race, and age [1]. Others include obesity, poor diet, lack of physical activity, and tobacco. It is the most familiar form of diabetes and has reached epidemic proportions in most parts of the world [3]. This could be aligned with current lifestyles [4]. The number of diabetic patients substantially increased between 1980 and 2014, and in 2014, 422 million people over the age of 18 were recorded as having diabetes [1]. In 2012, 1.5 million deaths were caused by diabetes, with the majority of cases T2DM [1]. Every year, 1.4 million Americans are diagnosed with diabetes, and if this trend continues, one in three Americans will suffer from diabetes by 2050 [5].

Medicinal plants have traditionally been consumed in different cultures to cure diabetes. The genus *Psychotria* has many applications in conventional medicine and provides tremendous potential for pharmacological properties. The genus *Psychotria* is traditionally known among the Karo people of North Sumatra as a treatment for diabetes, named “loning”; it is also called “meroyan sakat/salung” in Malaysia [6]. *Psychotria malayana* Jack is a plant of the Rubiaceae family and has several synonyms: *Grumilea aurantiaca*, *Pilosella aurantiaca*, *Chassalia expansa*, *Psychotria stipulacea*, and *Uragoga malayana*. Hadi et al. [7] reported the derivation of major alkaloids, hodgkinsine, and other compounds, including calycanthine, (+/−)-chimonanthine, meso-chimonanthine, 3-methyl-1,2,3,4-tetrahydro-γ-carboline, and 2-methyl-6-methyl pyrazine, from *P. malayana* leaves. Further, Hadi [8] studied the anti-bacterial activities of LPM-574, which is a derivative of hodgkinsine. Matsuura et al. [9] investigated the analgesic activities of hodgkinsine, (+)-chimonanthine, and meso-chimonanthine, whereas Chebib et al. [10] demonstrated the anti-convulsant effect of calycanthine. Despite scientific reports of the plant’s activities, data on its anti-diabetic impact are scarce.

Fourier Transform Infrared Spectroscopy (FTIR) is one of the most widely used techniques for the identification of chemical constituents and the elucidation of structural compounds. It has been used as a main method for identifying medicines in many countries [11]. FTIR analysis offers rapid fingerprint screening of the plant extracts [12]. Asokkumar et al. [12] indicated the application of FTIR in profiling natural herbs, including *Solanum torvum*, *Phyllanthus amarus*, *Phyllanthus maderaspatensis*, and *Senna auriculata.* In addition, Nagarajan et al. [11] carried out an FTIR study to detect the active functional groups present in garlic powder (*Allium sativum*).

No report of the correlation of the alpha-glucosidase inhibition (AGI) activity of the *P. malayana* leaf extract to its infrared spectrum has been made yet. In this study, therefore, an attempt is made to examine the bioactive compound functional groups that are present in the leaf extracts (in various solvents ratios of methanol-water) by using the FTIR technique. The present study attempted to develop a multivariate calibration model to predict the AGI activity of *P. malayana* Jack leaf extracts in correlation to their FTIR spectra.

The AGI enzyme suppresses glucose absorption by α-glucosidase enzyme (AGE) inhibition, which is an effective therapeutic strategy for the treatment of T2DM. The percentage of hydrolytic cleavage of oligosaccharides can be decreased by stopping the action of alpha-glucosidase, slowing the general rate of glucose absorption into the blood [13]. The goal of this work is to develop a calibration model able to evaluate metabolite fingerprint quality in relation to the FTIR spectrum.

## 2. Results and Discussion

### 2.1. Extraction Yield

The extraction yield of *P. malayana* leaf extracts is displayed in Table 1. The yield % of the solvent dilutions differed considerably from each other (*p* < 0.05). The 75% solvent ratio of methanol-water extracts of *P. malayana* resulted in the highest yield (35.19 ± 1.11%). In comparison, the 0% methanol-water extract was found to have the lowest percentage of yield (20.40 ± 1.47). The trend of this result was found as 75% > 50% > 100% > 25% > 0%. From this result, it is observed that to get a higher yield of extraction, a solvent of medium polarity is preferable to that of higher polarity. This result further indicated that a high proportion of methanol could be the perfect solvent for increasing extraction yields. This is in agreement with Khatib et al. [14], where a higher yield of extraction was related to the water–alcohol combinations rather than the monocomponent solvent. Sultana et al. [15] also reported that aqueous methanol is the most suitable solvent for extracting the leaves of a medicinal plant with a higher yield of extraction.

The choice of the solvents to be used in the present study is in line with the goal of this work. It requires a solvent that can extract a wide range of compounds, such as methanol. Once the quality of the sample has been evaluated through this rapid test, one can use ethanol or water to extract the plant for consumption purposes.

### 2.2. AGI Activity

Table 1 shows the AGI activity of *P. malayana* leaf extracts in various ratios of methanol-water. It is shown as the IC_50_ value in µg/mL, the minimum value representing the maximum inhibitory activity. There were no significant differences found in the inhibition potential of the 0% and 50% methanol-water extracts. In contrast, the 25%, 75%, and 100% methanol-water extracts exhibited notable differences (*p* < 0.05). The result shows that all extracts exhibited high AGI activity. The 100% methanol extract has the highest AGI activity with the IC_50_ value of 2.83 µg/mL, and taken together it was found that *P. malayana* leaf extract has notable AGI activity. In contrast, Jemain et al. [16] reported that *P. malayana* leaf showed only α-amylase inhibition (15.7 ± 0.8 µg/mL) and no α-glucosidase inhibition. This contrast could be due to the fact that they used dichloromethane as an extraction solvent following maceration, which is a technique for extraction. Due to the various absorbance conditions, methods of extraction and solvents used could alter the extraction yield and the plant bioactivity, as shown in a previous study [15,17,18,19,20].

### 2.3. FTIR Spectra of the P. Malayana Extracts

**Figure 1:**
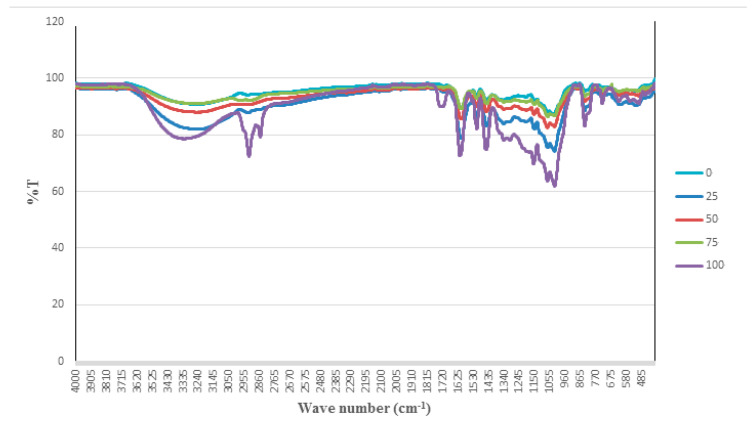
Infrared spectra of *Psychotria malayana* leaf extracts (0%, 25%, 50%, 75%, and 100% methanol-water).

FTIR is one of the most extensively used analytical techniques, not only used to identify chemical constituents but also to elucidate structural components [11]. FTIR spectra of different ratios of methanol-water extracts (0, 25, 50, 75, and 100% methanol) obtained from the *P. malayana* leaves are presented in Figure 1. As per the vibrational mode, the assigned peaks’ attribution of the *P. malayana* FTIR spectra are shown in Table 2.

Several functional groups, including alkanes, alkene, aromatics, and carbonyl groups, were found. Besides these, hydroxyl and amino groups were identified. The peak found for the bond stretch frequencies for O-H at 3010–3670 cm^−1^ indicates alcohol, phenol, and carboxylic acid groups. The C–H stretching vibrations in the alkanes, alkenes, aldehydes, and aromatic groups produced a strong band in the region of 2795–3010 cm^−1^. The peak found at 1666–1730 cm^−1^ indicates the existence of the carbonyl group (C=O), which means ester, carboxylic acid, and carbonyl compounds. Besides this, the peaks showed at 1550 to 1660 cm^−1^ and near 1500 cm^−1^, respectively, reveal the existence of alkenes (C=C) and secondary aromatic amines (N-H). C-N stretching indicating aromatic amines showed a strong band in the region of 1250–1350 cm^−1^, and C=C bending, which represents alkene, was observed in the region of 650–1000 cm^−1^. The peak found in the area of 430 to 800 cm^−1^ indicated alkanes and out-of-plane C-H bending of the aromatic end [21].

Based on the spectra, it was found that the peak representing C-H stretching was more prominent in the 100% methanol extract, whereas the same peak was absent in the other extracts (0, 25, 50, and 75% methanol). This observation indicates the existence of alkanes, alkenes, aldehydes, and aromatic compounds in the 100% methanol extract.

Besides that, the peak of C=O stretching was more significant in the 100% methanol extract than in the other extracts, which indicates the presence of ester, carboxylic acid, and carbonyl compounds in the 100% methanol extract. Furthermore, the peak indicating the presence of an amino compound was sharp in the 100% methanol extract, while in other extracts it appeared as a tiny shoulder, confirming the presence of aromatic amines [21]. Apart from this, peaks of C=C and C-H out-of-plane bending were found to be more significant in the 100% methanol extract in comparison with all other extracts. Some alpha-glucosidase inhibitors identified by gas chromatography–mass spectrometry (GC-MS)-based metabolomics were bearing the aforementioned functional groups related to the active compounds, including alpha-tocopherol (vitamin E), palmitic acid, beta-tocopherol, 1-monopalmitin, and stigmast-5-ene [22,23,24], which may be present in *P. malayana*.

Conclusively, gradient extraction solvents showed different effects in the yield as well as the bioactivity of the extract. Furthermore, the selectivity of the compounds and their bioactivity were also affected by variation in solvent polarities [15,17,18,19,20].

### 2.4. Multivariate Data Analysis (MVDA)

While most statistical techniques focus on just one or two variables, Multivariate data analysis allows more than two variables to be analysed at once. In MVDA, OPLS is one of the most familiar models. An impressive separation can be obtained by orthogonal partial least square–discriminant analysis (OPLS-DA) [25]. Wagner et al. [26] discussed the improved classification results based on the OPLS model. Yuliana et al. [27] also reported that OPLS is a suitable tool for studying the chemical profile–activity correlation.

**Figure 2:**
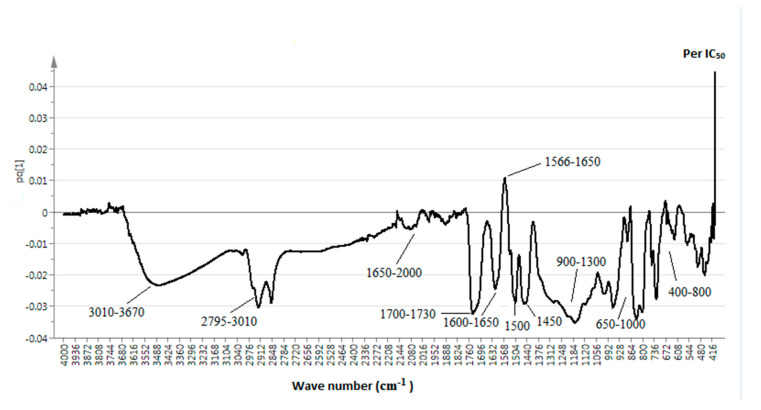
OPLS line loading plot of *P. malayana* Jack leaf extracts.

**Figure 3:**
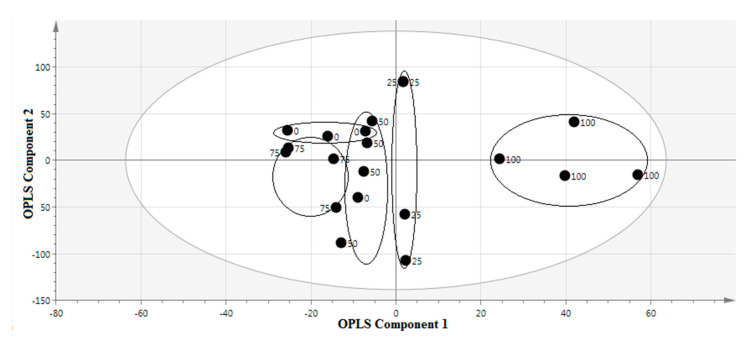
Orthogonal partial least square (OPLS) score plot of various solvent ratios of *P. malayana* Jack leaf extracts.

The discrimination of the samples employing the OPLS model is shown in Figure 2. In this OPLS model, the first matrix, considered as the x-variable, indicates the data acquired from the FTIR spectra. In contrast, the second matrix, considered as the y-variable, represents the per IC_50_ of AGI activity. This model shows two components (1 + 1 + 0) with a R^2^Y and a Q^2^Y (cumulative) of 65.9 and 50.5%, respectively. The samples having high AGI activity (100% and 25% methanol-water extracts) are on the positive side of the OPLS component 1 and differentiated from the extracts having less activity (75%, 50%, and 0% methanol-water extracts) that were clustered at the negative semicircle (Figure 3).

Table 3 shows the interrelation between the wave number from the FTIR spectra, considered as the x-variable, and the AGI activity (per IC_50_), considered as the y-variable. The plot exhibits the spectral data that influence the AGI activity. The peaks present at the negative axis of pq [1] of the plot correlate with the AGI activity and vice versa. The peaks found at 3010–3670 cm^−1^ and 2795–3010 cm^−1^, relating to the signals from the alpha-glucosidase inhibitors, showed bond stretch frequencies for O-H and C-H, respectively. These peaks indicate the presence of alcohol, alkanes, alkenes, aromatics, or aldehydes. Besides this, the peaks at 2000–1650 cm^−1^, 1700–1730 cm^−1^, and 1600–1650 cm^−1^ indicate C-H (bending), bond stretch frequencies for C=O, and bond stretch frequencies for C=C, respectively, which show the presence of aromatic compounds, carboxylic acid, and alkenes. Furthermore, the peaks found at 1450 cm^−1^ and 900–1300 cm^−1^ showed the presence of C-H bending and C-O (stretch) of secondary aromatic amines, an alkane, and alcohol, and ethers, esters, a carboxylic acid, and anhydrides, respectively. The presence of N-H bending was due to the appearance of a peak in the 1550 cm^−1^ region. Additionally, the peaks found at 650–1000 cm^−1^ and 430–800 cm^−1^ show C=C (bending) and a long chain of C-H (out-of-plane bending), respectively.

**Figure 4:**
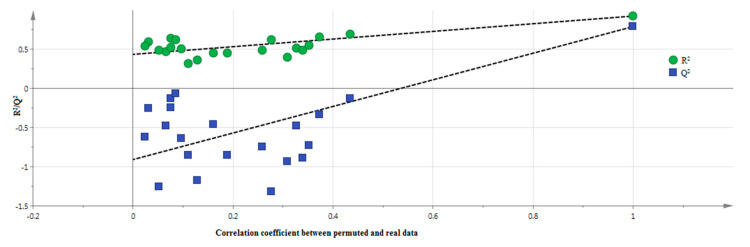
OPLS permutation plot of all *P. malayana* Jack leaf extracts.

Apart from this, the peaks present at the positive side of pq [1] of the plot (Figure 4) indicate the functional groups that do not make any contribution to the AGI activity. It shows the bond stretch frequencies for C=C at 1566–1650 cm^−1^ indicating the cyclic alkene. This finding can be a guideline for future bioactive compounds isolation.

Hadi et al. [7] identified major alkaloids, hodgkinsines, and other compounds, including calycanthine, (+/−)-chimonanthine, meso-chimonanthine, 2-ethyl-6-methylpyrazine, and 3-methyl-1,2,3,4-tetrahydro-γ-carboline from the leaves of *P. malayana.* Matssura et al. [9] illustrated the analgesic activities of hodgkinsine, (+)-chimonanthine, and meso-chimonanthine. Hadi [8] found the anti-bacterial activities of LPM-574, which is a derivative of hodgkinsine. Chebib et al. [10] reported the anti-convulsant effect of calycanthine.

### 2.5. MVDA Validation

The calibration model’s validation is vital for ensuring the authenticity of the predictive model. For the achievement of reliable data, it is crucial to overcome the risk of over-fitting the data. Model validation may be performed by cross-validation [28]. The ultimate predictive capacity of the model can be measured, and from this cross-validation the importance of the latent variable can be assessed [29].

**Figure 5:**
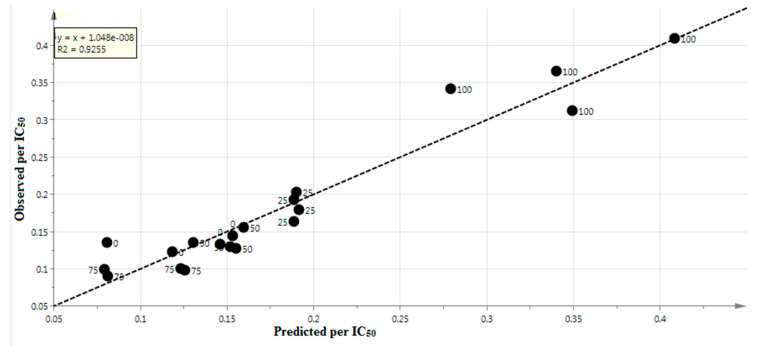
Observed versus predicted per IC_50_ data from *P. malayana* extracts.

From the obtained data shown in Figure 4, it can be concluded that the validity of the model developed is appropriate, because the total sum of the squares intercepting the Y-value and the predictive ability of the model intercepting the Y-value were less than 0.4 and 0.05, respectively [29]. In the present study, both of the model’s intercepting Y-values met the specifications. Its residuals were linear with an R^2^ of 0.9255, which represents the match between the experimental data and the model’s predictions (Figure 5).

Regarding the acceptability and predictability of the model, it is vital to include external samples [30]. A total of six external samples were extracted using 100% methanol, and Table 3 shows the original and predicted data for AGI activity. Based on the FTIR spectra, the AGI activity for all six samples was predicted and measured using the calibration model. All six external extracts showed high activity. Therefore, for predicting the AGI activity of *P. malayana* leaf extracts, the developed calibration model is valid.

## 3. Materials and Methods

### 3.1. Materials

Every single organic chemical purchased from Merck (Darmstadt, Germany) (methanol, dimethyl sulfoxide) was of analytical quality. Purchased standard quercetin was from Sigma-Aldrich (St. Louis, MO, USA); whereas the AGE, from yeast maltase, was obtained from Megazyme, Ireland. Besides this, the α-glucosidase (PNPG) was obtained from Sigma-Aldrich.

### 3.2. Collection and Preparation of Sample

The *P. malayana* plant was acquired from Cermin Nan Gedang in the district of Sarolangun, Jambi, Indonesia, and defined by a botanist, Shamsul Khamis. The plant sample was deposited at the KOP Herbarium, IIUM, Kuantan, for authentication. For seven days, the leaves were dried at ambient temperature and then powdered utilizing a universal cutting mill bought from Fritsch, Germany. The powdered plant leaves were preserved at −80 °C before extraction [22,31].

### 3.3. Preparation of Samples

About 400 g of *P. malayana* leaves were collected from six different areas of Indonesia for the purpose of validation. The leaves were cleaned and dried for seven days at ambient temperature. After that, the leaves were ground into powder utilizing a universal cutting mill (Fritsch, Germany) and preserved in a freezer having the temperature of −80 °C [22,31]. The crude extracts were prepared with 100% methanol after sonication for 30 min. AGI activity was determined for each extract, and FTIR analysis was used to evaluate the inhibitory activity of α-glucosidase utilizing multivariate data analysis [22,30].

### 3.4. Preparation of P. Malayana Extracts

For extraction, a common technique was adapted with approximately 1 g of plant powder. The extraction process was carried out through the sonication technique. The 1 g of plant powder was immersed in 30 mL of water and methanol at different ratios (0, 25, 50, 75, and 100% *v*/*v*) and sonicated for 30 min at 40 °C. The filtrate was then obtained utilizing Whatman^®^ grade one filter paper. A rotary evaporator was used at the temperature of 40 °C to remove any remaining solvents. Each extract was processed in four replicates, followed by storage at −80 °C before further use. For each of the five different methanol concentrations, a total of four replicates were prepared, resulting in 20 samples in total. Each extract of *P. malayana* was assessed for in vitro AGI activity and analyzed utilizing FTIR. The percentage of extraction yield was measured using the following formula:

Extraction yield (%, *w*/*w*) = Wt_a_/Wt_b_ × 100% (1)

where Wt_a_ indicates the final weight of the freeze-dried extract, and Wt_b_ is the initial weight of the raw plant powder [22].

### 3.5. AGI Assay

The in vitro AGI activity was determined following the assay of Javadi et al. [22]. Quercetin (positive control) is a well-known AGE inhibitor, prepared by dissolving 2 milligrams in 1 milliliter of dimethyl sulfoxide (DMSO). On the other hand, the substrate ρ-nitrophenyl-ρ-D-glucopyranoside (PNPG) is prepared by weighing 6 milligrams of the substrate and dissolving it in 20 milliliters of 50 millimolar phosphate buffer prior to the adjustment of the pH value to 6.5 using sodium hydroxide solution. The dried plant extracts were prepared in the same way as the quercetin. Ten microliters (10 µL) of quercetin (positive control), the samples, and DMSO (negative control) were added into a 96-well plate. One hundred microliters of 30 millimolar phosphate buffer and 15 microliters of the enzyme were added into the reaction mixture. The blank was the one without enzyme. After five minutes of incubation at ambient temperature, 75 microliters of PNPG was added to the samples and the blank mixture. After another 15 min of incubation, the catalytic reaction was halted by the addition of 50 microliters of glycin. The absorbance was taken at 405 nm utilizing a microplate reader (Tecan Nanoquant Infinite M200, Tecan, Männedorf, Switzerland). The IC_50_ was calculated from the linear regression analysis. IC_50_ is an important tool for measuring the efficacy and potency of an inhibitor; it represents the amount of inhibitor needed to halve the response [32]. All determinations were performed in triplicate. The AGI activity (%) was determined using the formula below:

Inhibitory activity (%) = [(A_control_ − A_sample_)/A_control_] × 100% (2)

where A_control_ indicates the negative control’s absorbance, and A_sample_ represents the sample’s or positive control’s absorbance [33].

### 3.6. FTIR Analysis

The experiment was carried out with an FTIR spectrometer (Perkin Elmer Inc., Waltham, MA, USA) fitted with a horizontally attenuated total reflectance system with a diamond crystal. The instrument was adjusted to ambient temperature before operation. A minimal quantity of each freeze-dried sample was put onto the diamond crystal using a washed spatula [34]. FTIR spectra were assessed in the wave region of 400–4000 cm^−1^ and at the resolution of 4 cm^−1^. The Attenuated total reflection (ATR) crystal was cleansed with utmost care between measurements. The data were collected and processed through the software Perkin Elmer Spectrum version 10.03.09 (Waltham, MA, USA). The data were then measured using multivariate data analysis [30].

### 3.7. Statistical Analysis

All data are shown as mean ± standard deviation (SD) utilizing Minitab 17 (Minitab Inc., State College, PA, USA). One-way analysis of variance (ANOVA) along with a Tukey’s comparison test were used to assess the differences, which were considered significant at *p* < 0.05 with a confidence interval of 95%. In the interim, the spectra acquired from the infrared assay were changed into ASCII format. For multivariate data analysis (MVDA), the data were then translated into Microsoft Excel format and brought up in the Simca P^+^ 14.0 software (Umetrics, Umeå, Sweden) using an orthogonal partial least square (OPLS) model for the AGI activity and the spectral data.

## 4. Conclusions

This research explored the potential anti-diabetic activity of *P. malayana* leaf extracts at different methanol-water ratios (0 %, 25 %, 50%, 75 %, and 100% methanol-water). The 100% methanol-water extract showed the maximum inhibitory activity. The correlation of FTIR spectra and AGI activity identified the functional groups that are able to induce AGI activity, namely hydroxyl (O-H), alkenyl (C=C), methylene (C-H), carbonyl (C=O), and secondary amine (N-H) groups. This analysis has developed a validated statistical model, with an R^2^Y value of 0.9255 indicating the fitness of the model. The AGI activity of *P. malayana* was predicted using this validated statistical model. This finding shows that the developed model is statistically valid to predict AGI activity, which is very useful as a tool in the quality control of this plant.

## Figures and Tables

**Table 1:** Alpha-glucosidase inhibition (AGI) activity (IC_50_) and the percentage of yield of *Psychotria malayana* leaf extracts.

Ratios of Methanol and Water.	AGI Activity IC_50_ (µg/mL)	Percentage of Yield
0/100	7.48 ± 0.51 ^b^	20.40 ± 1.47 ^d^
25/75	5.45 ± 0.51 ^c^	28.67 ± 1.01 ^c^
50/50	7.35 ± 0.66 ^b^	32.73 ± 1.41 ^a,b^
75/25	10.34 ± 0.52 ^a^	35.19 ± 1.11 ^a^
100/0	2.83 ± 0.32 ^d^	31.51 ± 1.24 ^b^
Quercetin	1.86 ± 0.04 ^d^	ND

The data that do not have a similar letter are notably different (*p*-value < 0.05). With multiple superscripts, the values were calculated using Tukey’s multiple comparison test. Results are presented as mean ± standard deviation. ND = not determined. table solvent for higher yield of extraction. Sultana et al. also reported that aqueous methanol is the most suitable solvent.

**Table 2:** Functional groups of *P. malayana* leaf extracts as seen by Fourier Transform Infrared Spectroscopy (FTIR) analysis [21].

Region of Wavenumber (cm^−1^).	Functional Group	Name of Functional Group Attribution
3010 to 3670	O-H stretch	Phenols, alcohols, and carboxylic acid
2795 to 3010	C-H stretch	Aromatics/aldehydes, alkanes, and alkenes
1666 to 1730	C=O stretch	Aldehydes/primary amides, carboxylic acid, ester, and ketone
1550 to 1660	C=C	Alkenes
1300 to 1390	N=O	Aliphatic nitro compounds
650 to 1000	C=C bend	Alkenes
430 to 800	C-H	430 to 800

N = nitrogen; O = oxygen; H = hydrogen; C = carbon.

**Table 3:** The actual and predicted AGI activity of the 100% methanol extract of the external samples of *P. malayana* leaves.

Number of Sources	Actual AGI Activity IC_50_ (µg/mL)	Predicted AGI Activity
1	5.70 ± 0.44 ^a^	High Activity
2	5.58 ± 0.38 ^a^	High Activity
3	2.63 ± 0.35 ^c^	High Activity
4	3.80 ± 0.81 ^b,c^	High Activity
5	5.10 ± 0.70 ^a,b^	High Activity
6	5.60 ± 0.66 ^a^	High Activity

Results are presented as mean ± standard deviation.The data that do not have a similar letter are notably different (*p*-value < 0.05). With multiple superscripts, the values were calculated and represented using Tukey’s multiple comparison test.
